# CD4^+^ T cell activation and inflammation in NASH-related fibrosis

**DOI:** 10.3389/fimmu.2022.967410

**Published:** 2022-08-10

**Authors:** Yunfeng Zhou, Haibo Zhang, Yao Yao, Xiaoyan Zhang, Youfei Guan, Feng Zheng

**Affiliations:** ^1^ Department of Physiology, Medical Research Center, Shenzhen University, Shenzhen, China; ^2^ Advanced Institute for Medical Sciences, Dalian Medical University, Dalian, China; ^3^ Division of Nephrology, Affiliated Hospital of Nantong University, Nantong, China; ^4^ Wuhu Hospital & Health Science Center, East China Normal University, Shanghai, China

**Keywords:** Liver fibrosis, NASH, innate immune response, adaptive immunity, CD4^+^ T cells

## Abstract

Liver fibrosis is a common pathological feature of end stage liver failure, a severe life-threatening disease worldwide. Nonalcoholic fatty liver disease (NAFLD), especially its more severe form with steatohepatitis (NASH), results from obesity, type 2 diabetes and metabolic syndrome and becomes a leading cause of liver fibrosis. Genetic factor, lipid overload/toxicity, oxidative stress and inflammation have all been implicated in the development and progression of NASH. Both innate immune response and adaptive immunity contribute to NASH-associated inflammation. Innate immunity may cause inflammation and subsequently fibrosis *via* danger-associated molecular patterns. Increasing evidence indicates that T cell-mediated adaptive immunity also provokes inflammation and fibrosis in NASH *via* cytotoxicity, cytokines and other proinflammatory and profibrotic mediators. Recently, the single-cell transcriptome profiling has revealed that the populations of CD4^+^ T cells, CD8^+^ T cells, γδ T cells, and TEMs are expanded in the liver with NASH. The activation of T cells requires antigen presentation from professional antigen-presenting cells (APCs), including macrophages, dendritic cells, and B-cells. However, since hepatocytes express MHCII molecules and costimulators, they may also act as an atypical APC to promote T cell activation. Additionally, the phenotypic switch of hepatocytes to proinflammatory cells in NASH contributes to the development of inflammation. In this review, we focus on T cells and in particular CD4^+^ T cells and discuss the role of different subsets of CD4^+^ T cells including Th1, Th2, Th17, Th22, and Treg in NASH-related liver inflammation and fibrosis.

## Introduction

With the high prevalence of obesity, diabetes, and metabolic syndrome worldwide, the morbidity of nonalcoholic fatty liver disease (NAFLD) is rapidly increased (1, 2). NAFLD may evolve from simple steatosis to nonalcoholic steatohepatitis (NASH), which may further progress to liver fibrosis and cirrhosis (3). NAFLD now represents the most common liver metabolic disease all over the world. It is predicted that by 2030, more than 300 million peoples in China, 100 million in the USA, and 15-20 million in the major European countries will suffer from NAFLD (4). Moreover, the number of NASH patients in the USA will reach 27 million by 2030 (4). The prevalence of NAFLD/NASH has increased from 23.8 to 32.9% in China during 1998-2018 (5), with the total number of NASH patients in China reaching 48.26 million by 2030 (4). Hepatic fibrosis is an independent predictor of disease related mortality in NASH. The fatality rate in NASH-related cirrhosis ranges from 12 to 25% (6). NASH has become the leading causes for liver transplantation in the developed countries (6). From 2004 to 2016, the registration number of liver transplantation resulted from NASH was increased by 114% in males and 80% in females (7). Unfortunately, the pathogenic mechanisms underlying NASH remains unclear and the effective new drug(s) and therapies for the disease are urgently needed.

NASH is characterized by the presence of hepatic steatosis, hepatocellular damage, inflammation, and varying degrees of fibrosis, subsequently progressing to cirrhosis and end-stage liver disease (1, 6). A large body of evidence demonstrates that lipotoxicity, oxidative stress and inflammation act in concert in promoting the pathogenesis of NASH and liver dysfunction. If the liver fails to repair in the event of persistent injury, progressive fibrosis and functional decline occur (8–12). Metabolic dysfunction such as hepatic steatosis is considered an early event in the pathogenesis of NASH. Excessive accumulation of fat (lipotoxicity) in the liver not only constitutes the first hit in the disease, but also causes hepatocyte injury and liver insulin resistance and inflammation, contributing to disease progression. Currently known lipids with liver toxicity include saturated fatty acids, diacylglycerols, ceramide, free cholesterol (FC), and sphingomyelin (SM). It is generally believed that among many pathological factors, lipotoxicity-elicited, innate and adaptive immunity-mediated inflammation plays a central role in the development and progression of NAFLD/NASH.

## Innate immunity in NAFLD/NASH

NLRP3 is an important component involved in the innate immunity, which functions as a pattern recognition receptor (PRR) that senses both pathogen- and danger-associated molecular patterns (13). NLRP3 is highly expressed in the Kupffer cells, where its activation significantly aggravates NASH by secretion of pro-inflammatory cytokines, such as IL-1β and IL-18 (14). In contrast, palmitic acid-induced inflammation in the Kupffer cells is reduced and NASH development is prevented in the NLRP3^-/-^ mice (15). Similarly, deficiency of NLRP3 protects mice from liver macrophage infiltration and activation and attenuates liver injury and fibrosis (16).

Kupffer cells (KCs), the predominant tissue-specific resident macrophages in the liver (17–19), are situated on the liver sinusoids and lymph nodes (19). The primary function of the KCs is to remove pathogens or bacteria-derived toxins and debris, generating innate immune response. Depletion of hepatic KCs by clodronate liposomes or gadolinium chloride alleviates liver steatosis and inflammation in high-fat diet (HFD)-induced fatty liver animal models, suggesting an essential role of the KCs in NAFLD/NASH (20, 21). Additionally, the C-C motif chemokine receptor 2 (CCR2^+^) monocytes, which are derived from bone marrow and recruited to the liver by CCR2, are crucial in contributing to hepatic fibrosis, since their inhibition has been reported to ameliorate NASH (22). The KCs can also act as a professional antigen-presenting cells (APCs) to present antigen to T cells which are essential in the adaptive immunity (23). Proinflammatory macrophages were found to be significantly increased in the periportal zone in the livers of NASH patients and correlated with the severity of liver fibrosis (22). An additional mechanism by which the activation of KCs contributes to the development of NASH is the activation of local immune system and inflammatory response through energizing PRRs. The Toll-like receptor (TLR) family is one of major classes of the PRRs that play an essential role in the initiation of innate immune response. The roles of hepatic TLR2, TLR4 and TLR9 in NASH has been repeatedly reported (24–26). Activation of the TLR4 by LPS or TLR-9 by DNA derived from intestinal bacteria promotes steatohepatitis, while suppression of the TLR4 or TLR-9 attenuates liver steatosis, inflammation, and fibrosis in a few of mouse models of NASH (25, 27). Altogether, these findings demonstrate that activation of the NLPR3 inflammasome and TLRs may contribute to the development and progression of NASH.

Besides being a critical metabolic organ to controls body glucose and lipid metabolism, the liver is also an important immunological organ in inflammatory and immune response. In the past decade, a great progress has been made regarding how the immune cells are reshaped in the livers of animals and patients with NASH (28–31). However, the exact cellular composition of normal and steatotic livers in animals and humans remains incompletely understood. Since single-cell transcriptome analysis is very useful in uncovering the compositions and the numbers of immune cells as well as their differentiation and activation states in the livers, we utilized publicly available single-cell transcriptome databases and analyzed the types and numbers of hepatic immune cells between mice and humans (**Supplemental Figure 1**). We found that the percentages of CD4^+^ T cells, CD8^+^ T cells, NK T cells, NK cells, γδ T cells, TEMs, and monocytes in murine livers were less than those in humans, but the number of B cells, dendritic cells (DCs), and KCs were more in mice than those in humans (**Supplemental Figure 1**). Recent study has indicated that compared to controls, the mice with NASH exhibited increased proportions of hepatic CD4^+^ T cells, CD8^+^ T cells, γδ T cells, TEMs, B cells, DCs, and LAM, with reduced proportions of hepatic NK T cells, NK cells, monocytes and KCs (28). These findings demonstrate that in addition to innate immunity, adaptive immunity also plays a critical role in the pathogenesis of NAFLD/NASH. Below we will discuss the consequence of the changes in hepatic immune cell infiltration, with a focus on the role of T cell-mediated acquired immunity in NAFLD/NASH.

## T cell-mediated adaptive immunity in NASH

### General roles of T cells in NASH

T cells represent a major type of lymphocytes in the immune system and play a crucial role in the adaptive immune response. T cell clone can recognize antigen by the presence of a T cell receptor (TCR) on its cell surface. According to the differential physiologic functions, T cells can be subdivided in conventional T cells and innate-like T cells (unconventional T cells). Conventional T cells can be further classified into CD8^+^ cytotoxic T (Tc) cells and CD4^+^ T helper (Th) subsets, and innate-like T cells are composed of natural killer T (NKT) cells, γδ T cells and mucosal-associated invariant T (MAIT) cells (32, 33).

### Roles of antigen-presenting cells in NASH

It is well known that the major histocompatibility complex (MHC) is essential for specifically recognizing antigen by T cells. The MHC family includes MHCI and MHCII. The function of the MHCI molecules is to display intracellular proteins to CD8^+^ T cell, named as cytotoxic T cells (CTLs), while the MHCII molecules are highly expressed in antigen-presenting cells (APCs) to induce CD4^+^ T cell activation. APCs are divided into professional APCs and non-professional APCs. Professional APCs expressing the MHCII molecules include macrophages, dendritic cells (DCs), and B-lymphocytes. As mentioned above, the Kupffer cells (KCs), a kind of specified hepatic macrophages, are critically involved in the development and progression of NASH. The DCs act as a bridge between the innate and the adaptive immune responses (34). Hepatic DCs are mainly localized at the portal vein, with a minor presence at the central vein (35) and their numbers are markedly increased in NASH patients (36). An increase of conventional dendritic cells (cDCs) and cDC1s specifically presenting XCR1 was observed in NASH patients and models (37). CD11c^+^ cells have been found to exert a defensive effect on methionine and choline-deficient diet (MCD)-induced liver fibrosis (38). Moreover, there is a significant correlation between the circulating and hepatic cDC1 cell numbers and the severity of NASH (37). NASH was found to be associated with increased proliferating cDC1 progenitors. Specific depletion of cDC1s attenuates steatohepatitis in NASH mice (37), suggesting that cDC1s contribute to the pathogenesis of NASH. However, contradictory results also exist regarding the role of DCs in NASH. For example, no significant impact of DCs on the development of hepatic fibrosis was observed in bile duct ligation (BDL)- and carbon tetrachloride (CCl4)- induced NASH models (39). Thus, further studies are needed to clarify the role of different DCs in the pathogenesis of NASH.

B-lymphocytes, also known as B cells, also represent a classic type of leukocytes and the major humoral immunity component involved in adaptive immune response. B cells present essential immunological functions, such as producing antibody, presenting antigen, and secreting cytokines (40–42). The numbers of hepatic B cells in mice are much higher than those in humans (43) (**Supplemental Figure 1**). An accumulation of B cells is evident in the livers of NASH patients, which is accompanied by marked hepatic inflammation and fibrosis (44). Similarly, activated intrahepatic B cells were found to be markedly increased in NASH mouse models. Moreover, B cell deficiency can significantly ameliorate NASH phenotypes in mice, possibly because both B cell receptor-mediated adaptive immune signaling and myeloid differentiation primary response 88 (MyD88)-dependent innate immune response are involved in pathological actions of B cells on NASH (45). It is also noted that NASH is associated with altered gut microbiota and increased intestinal permeability. Thus, hepatic B cells may be inappropriately activated in a microbiota-dependent manner to participate in NASH inflammation (45).

Increasing evidence shows some other cell types expressing the MHCII molecules are capable of presenting antigen as atypical APCs, including the mast cells, basophils, eosinophils, neutrophils, innate lymphoid cells (ILCs), endothelial and epithelial cells (46). Our recent research showed that renal proximal tubule epithelial cells (PTECs) also represent as an atypical APC, which may promote the proliferation of CD4^+^ T cells in a MHCII-dependent manner (47). Emerging evidences demonstrate that the MHCII molecules are expressed in mouse and human hepatocytes (48–50). The levels of MHCII are increased in the hepatocytes of viral hepatitis and autoimmune hepatitis (48, 51). Compared to healthy control, there was higher expression of MHCII in the liver biopsies of NASH patients. Significantly increased levels of MHCII were also observed in the liver samples of patients with alcoholic hepatitis (AH), with marked upregulation of CD4 expression closely associated with MHC II producing hepatocytes in AH biopsies (52), suggesting that hepatocytes may function as important nonclassic APCs in NASH-related liver fibrosis.

### TCR and costimulatory molecules

TCR recognition of antigen as peptides bound to the MHC molecules provides the primary signal for T cell activation (53). The TCRs are comprised of two different heterodimers: TCRα/TCRβ or TCRγ/TCRδ (54). In the majority (95%) of T cells the TCRs consist of TCRα and TCRβ isoforms. αβ T cells were regularly referred to as T cells. However, a small proportion (less than 5%) of T cells (γδ T cells) are composed of TCRγ and TCRδ isoforms (54). Antigen recognition is achieved through the TCR-CD3 complex. CD3 is an essential T cell co-receptor, which is required for T cell activation. Recent study has showed that there is a marked decrease in TCR clonotypes (TCR TCRα, TCRβ, and TCRαβ) in CCl4-induced fibrotic livers (55). Furthermore, TCRβ gene knockout mice showed an aggravated hepatic fibrosis phenotype compared with WT mice, which is associated with the activation of hepatic stellate cells (HSCs) due to the expansion of macrophage and γδ T cells (55). These results indicate that TCR-mediated T cell activation may play an important role in the pathogenesis and progression of liver fibrosis.

Additionally, the full activation of CD4^+^ T cells requires a second co-stimulatory signal (56, 57). Costimulatory molecules are present on the surface of T cells and APCs binding with each other in a paired ligand-receptor manner, which leads to the activation of these cells and thus triggers immune response (58). OX40 and its ligand, OX40L are the members of the TNF receptor superfamily and produce a potent costimulatory signal that enhances T cell activation, proliferation, and differentiation (59). Recent study indicates that OX40 plays an essential role in regulating both liver innate and adaptive immunity and promotes NASH development and progression (60). Compared with the wild-type (WT) mice, OX40 global knockout (KO) mice exhibited an ameliorated NASH phenotype. Mechanistically, OX40 global deficiency suppresses Th1 and Th17 differentiation and inhibits monocyte migration during NASH development. Plasma OX40 levels were found to be positively correlated with NASH in patients, suggesting that OX40 may represent a diagnostic parameter and therapeutic target in NASH (60). Together, these studies have indicated that T cell costimulatory molecules contribute to the development and progression of NASH. However, it is still uncertain whether hepatocytes supply the T cell with costimulatory signal to activate and drive inflammation. The expression of OX40L on hepatocytes in mice is undetectable, although they are expressed on other hepatic APCs, such as KCs and DCs (61). Thus, further studies are needed to address these unanswered questions.

### The roles and mechanisms of CD4^+^ T cells in NASH

T helper cells, as known as CD4^+^ T cells, are involved in immune processes and express membrane surface marker CD4 (62). Dysfunction of CD4^+^ T cells is emerging as an important pathological factor engaged in the progression of NAFLD and NASH. An accumulation of peripheral and intrahepatic CD4^+^ T cells was revealed in human and mouse NASH models (63–65). In a study in which human T cells were transferred to NOD-scid IL2rg^null^ (NSG) mice to identify human-specific immune response in NASH, CD4^+^ T cells were found to be crucial in promoting liver steatosis-fibrosis transition (65). Moreover, *in vivo* depletion of human CD4^+^ T cells can efficiently reduce proinflammatory cytokine production and fibrosis in the humanized NASH mice, further confirming the importance of CD4^+^ T cells in the pathogenesis of NASH (65). Other evidence also supports a potential role of CD4^+^ T cells in promoting NASH by releasing proinflammatory cytokines, because MCD-HFD-induced NASH can be significantly attenuated in mice deficient for IFNγ (66). It is well known that CD4^+^ T cells have several functionally diverse subsets, such as Th1, Th2, Th17, Th22, and regulatory T cells (Tregs), which are characterized by expression of different cytokines respectively (67, 68). Although overall CD4^+^ T cells are critically involved in NASH-related inflammation and fibrosis, the role and mechanism of each CD4^+^ T cell subset in the onset and progression of NASH may be different and are summarized in **Table 1** and discussed in the following sessions.

**Table 1:** Roles of diverse CD4^+^ T cell subsets in NASH.

Cell subset	Effect	Mechanism	References
Th1 cells	Profibrotic	IFN-γ dependent manner	(66, 69, 70)
Th2 cells	Complicated	Production of type 2 cytokines *via* IL-33	(71, 72)
Th17 cells	Profibrotic	An IL-17–driven fibrotic process	(73–75)
Th22 cells	Bidirectional	Production of IL-22	(76, 77)
Treg cells	Antifibrotic(mainly)	Immunosuppression by secretion of IL-10	(78, 79)

Different CD4^+^ T cell subsets exhibit diverse effects on liver fibrosis. Th1 and Th17 cells are proinflammatory and profibrogenic while the role of Th2 cells in hepatic fibrosis is complicated. Th22 and Treg cells may be both anti- and/or pro-fibrotic depending on disease setting and the stage of the disease.

### TH1 cells and liver fibrosis

T helper 1 (Th1) cells exhibit proinflammtory effects *via* expressing the transcription factor T-bet and producing cytokine IFN-γ, IL-2 and TNF-α through the activation of STAT4 and STAT1 (80). Compared to healthy controls, there was an elevation in Th1 cell proportions in peripheral blood of NAFLD and NASH patients although there were not differences in Th1 cell numbers in peripheral bloods and hepatic tissues between NAFLD and NASH patients (64). Nevertheless, there is an increase of genes toward Th1 phenotype in NSAH compared with NAFLD patients (81). In animals, hepatic Th1 cells were found to be increased in a MCD diet-induced mouse NASH model (82). Since IFN-γ is produced by Th1 cells, IFN-γ gene KO mice are applied to determine the role of Th1 cells in NASH. IFNγ-deficient mice exhibit less steatohepatitis and attenuated fibrosis than wild-type (WT) littermates with an MCD-high-fat diet (66). These results are indirectly supported by clinical observations that both pediatric and adult NASH patients have elevated circulating and hepatic IFN-γ-producing CD4^+^ T cells (69, 70). Consistently, an upregulation of hepatic Th1-related cytokine IFN-γ, IL-12 and TNF-α was observed in steatotic mice induced by concanavalin A (CoA) hepatitis plus choline-deficient diet, which was accompanied with an increase in T-bet and STAT4 expression (83). Collectively, these findings demonstrate that Th1 cells exert proinflammatory and profibrotic effects on NASH, probably by an IFN-γ dependent manner.

### TH2 cells and liver fibrosis

In general, Th2 cells exert an anti-inflammatory effect to ensure a protective immune response (82). Th2 cells are characterized by the transcription factor (TF) GATA3 and dominantly produce cytokine IL-4, IL-5, and IL-13 by the activation of STAT5 and STAT6 (78, 84). Several studies have supported a role of Th2 cells in NASH. Compared to healthy normal controls, an increase in peripheral blood Th2 cells in NAFLD patients was observed (64). Moreover, the Th2/Treg ratio in peripheral bloods was significantly increased in NAFLD patients, and was markedly decreased in NAFLD patients after 12 months bariatric surgery. However, there is not difference in Th2/Treg ratio in either peripheral blood or the liver between NASH and NAFLD patients (65). Serum levels of Th2 cytokine IL-13 were found to be elevated in NASH patients, accompanied by increased hepatic expression levels of its receptor IL-13Rα2 (71). It has been reported that functional IL-13Rα2 was upregulated in activated hepatic stellate cells (HSCs) in NASH, and IL-13 cytotoxin-mediated killing of IL-13Rα2^+^ cells can ameliorate liver fibrosis in a rat model of NASH, indicating the involvement of the IL-13/IL-13Rα2 pathway in NASH (71). Since IL-33 can promote Th2 response and increase the production of type 2 cytokines, such as IL-4, IL-5, and IL-13, which leads to extracellular matrix accumulation, administration of recombinant IL-33 to mice exaggerated liver fibrosis in NASH mice (72). However, IL-33 at the same time decreased hepatic triglyceride storage and reduced liver injury (72), suggesting that the role of IL-33 in NASH is complicated. Therefore, the contribution of Th2 cell-mediated adaptive immunity to NASH remains inconclusive and needs to be further defined.

### TH17 cells and liver fibrosis

T helper 17 (Th17) cells are commonly known as proinflammatory cells and characterized by specific expression of active TF retinoic acid receptor-related orphan receptor γt (RORγt) and STAT3 (85). Th17 cells mainly produce IL-17, IL-22 and IL-23. The IL-17 family is composed of six members namely IL-17A-F (86). An increase in the number of Th17 cells was repeatedly observed in the livers of NAFLD/NASH animal models (82, 87–90). Moreover, increased number of Th17 cells in circulation and the liver is also observed in NAFLD/NASH patients, accompanied with increased Th1 cells (64). However, the role of Th17 cells in the progression of liver fibrosis is uncertain. Several studies have showed an elevation in hepatic steatosis by administering IL-17, as well as an attenuation in liver fibrosis when blocking IL-17 (82, 88, 91, 92). However, there are other studies reporting an opposite effect in which enhanced liver steatosis was observed after functionally blocking IL-17 (89, 93). Th17 cell can induce hepatic inflammation possibly due to the accumulation of macrophages by IL-17-dependent elevation of chemokine CXCL10 (85, 89). It has been previously reported that IL-17 and IL-22 exhibit opposite effects in the development of NASH (86). For instance, IL-17 can increase, while IL-22 can prevent, palmitate-induced lipotoxicity to hepatocytes (82). Taken together, Th17 cells promote hepatic inflammation and fibrosis possibly by acting on liver cells particularly the Kupffer cells and Stellate cells to accelerate the fibrotic process (73–75).

### TH22 cells and liver fibrosis

T helper 22 (Th22) cells are specified by producing IL-22 in the absence of IL-17 (94). The differentiation of Th22 cell is promoted by IL-6 and TNFα, and hindered in the presence of TGFβ. Activation of the transcription factor aryl hydrocarbon receptor (AhR) markedly promotes Th22 cells to produce IL-22 (94). IL-22 may exert an inhibitory effect on the development of NAFLD. In animals, hepatic steatosis was markedly attenuated and transaminase levels were significantly reduced by the adminiatration of recombinant IL-22, possibly *via* a STAT3-mediated mechanism (76, 77). In addition, short-term IL-22 treatment is capable of decreasing hepatic expression of PPARα, PPARγ, and SREBP-1c, while long-term treatment is able to decrease the expression of hepatic fatty acid synthase (FAS) and very long chain fatty acids protein 6 (ELOVL6) (77). It has been reported that IL-22 can attenuate palmitate-induced lipotoxicity in a PI3K/Akt-dependent manner to inhibit JNK, which may explain why IL-22 downregulates transaminase levels. Intriguingly, IL-22-mediated hepatoprotection was only effective in the absent of IL-17, which increases the expression of PTEN, a PI3K/Akt inhibitor (82). Collectively, these findings indicate that IL-22 can exert an antifibrotic effect, which may be beneficial in NASH. However, it has been reported that IL-22 treatment may increase the risk of hepatocellular carcinoma possibly by the activation of STAT3, which limits its clinical use as a therapeutic agent for NASH (95).

### Regulatory T cells and liver fibrosis

Regulatory T (Treg) cells play critical roles in modulating immune homeostasis. Tregs are defined by the expression of the transcription factor forkhead box P3 (Foxp3). Tregs exert their immunosuppressive effects by secreting the cytokine IL-10, and interfering with T-cell survival by IL-2 depletion to inhibit APCs maturation and functionality (78). The differentiation of Treg cells is driven by TGFβ in the absent of IL-6 and further augmented by IL-2- and retinoic acid-induced STAT5 activation (96). IL-6 is an important determinant that balances the differentiation between the Treg and Th17 (96). Studies showed that the function of Treg in visceral adipose tissue is PPARγ dependent (97). PPAR-γ is a major driver in the accumulation and the phenotype of Treg cells in adipose tissue. It has been reported that Treg cells lacking PPAR-γ exhibited a phenotype of insulin resistance, and the PPARγ agonist pioglitazone failed to restore its insulin sensitivity in Treg-specific PPARγ^-/-^ mice (97). Moreover, pioglitazone treatment can ameliorate HFD-induced hepatic steatosis and increased Treg cell numbers in the visceral adipose tissue and the liver (98).

A decrease in hepatic Treg cell numbers was observed in animal models of NAFLD (90, 99, 100), with the mechanisms accounting for decreased Treg cell numbers in NAFLD largely unknown. In steatotic livers, excessive oxidative stress leads to the apoptosis and reduction of hepatic Treg cells, which can be prevented by the antioxidant MnTBAP (99). Depletion of hepatic Foxp3^+^CD4^+^CD25^+^ Tregs may result in steatosis if the animals are fed with a high-fat diet, while reconstitution of Treg cells can attenuate the NASH phenotype, accompanied by the reduction of hepatic inflammation as evidenced by a downregulation in hepatic TNFα expression (99). Another study also showed that adoptive transfer of induced Tregs can alleviate the pathological (liver steatosis) and metabolic (high levels of blood glucose, cholesterol, and liver enzymes) abnormalities in leptin-deficient ob/ob mice, supporting a potential immunological approach for treatment of diabetes and steatosis by the induction of Tregs (101). Moreover, circulating and hepatic resting Treg cell numbers are lower in NAFLD patients than healthy controls, with even more robust reduction in patients with NASH (64). Thus, the liver Th17/resting Treg ratio may be useful in distinguishing patients with NASH from those with simple steatosis. Unlike resting Treg cells, although there is an increase in the circulating levels of the activated Tregs, the change in the numbers of activated Tregs in the liver remains controversial in NAFLD (102). Thus far, most available evidence demonstrates that Treg cells are antifibrotic at least in part due to its immunosuppressive effect through secretion of IL-10 (79). In fact, in a bile duct ligation animal model, depletion of Tregs exacerbates liver fibrosis, which is associated with a marked changes in IL-6 and IL-10 production (103). However, since Treg cells also secret TGFβ, which is widely regarded as an important profibrotic factor for the development and progression of liver steatosis and fibrosis (96, 104–106), Treg cells may have a dual role in NASH owing to their spatial and temporal actions in the process of the disease.

### γδ T cells and liver fibrosis

In additional to CD4 T cells, there is a significant proportion of γδ T cells in liver, responsible for 15%-25% of total T cells and 3-5% of total lymphocytes (107). Importantly, γδ T cells were found to be significantly increased in NASH in both humans and mice. γδ T cell population is an exclusive subset of CD3^+^ T cells characterized by a T cell receptor (TCR) γ chain and δ chain, and does not require MHC-mediated antigen presentation. γδ T cells may function as a connection between the innate and adaptive immunity because they express TCRγδ that recognizes certain antigens and also secrete pro-inflammatory cytokines such as IL-17A upon stimulation (108). In HFD- or high-fat/high-carbohydrate diet (HF/HCD)-induced NASH mice, a marked elevation in both adipose tissue and liver γδ T cells were observed, associated with liver steatosis, damage, and cirrhosis (109). Furthermore, γδ T cell Tcrd^-/-^ mice exhibited a significant attenuation in steatohepatitis compared to WT mice after HFD treatment. Transfer of HF/HCD-treated mice with WT hepatic γδ T cells, but not with IL-17A^-/-^ hepatic γδ T cells, exacerbated NASH in Tcrd^-/-^ mice, suggesting that hepatic γδ T cells may contribute to NASH progression (109). It has been reported that hepatic γδ T-cell infiltration is increased in a CCR2-dependent manner in three animal models of steatohepatitis, including alcoholic steatohepatitis (ASH), MCD-induced NASH, and HFD plus ethanol-induced model (110). Depletion of γδ T cells can reduce liver steatosis, leukocyte infiltration, and inflammation (110), possibly by inhibiting the innate and adaptive immune responses during NASH progression (111).

## Conclusion and perspectives

**Figure 1:**
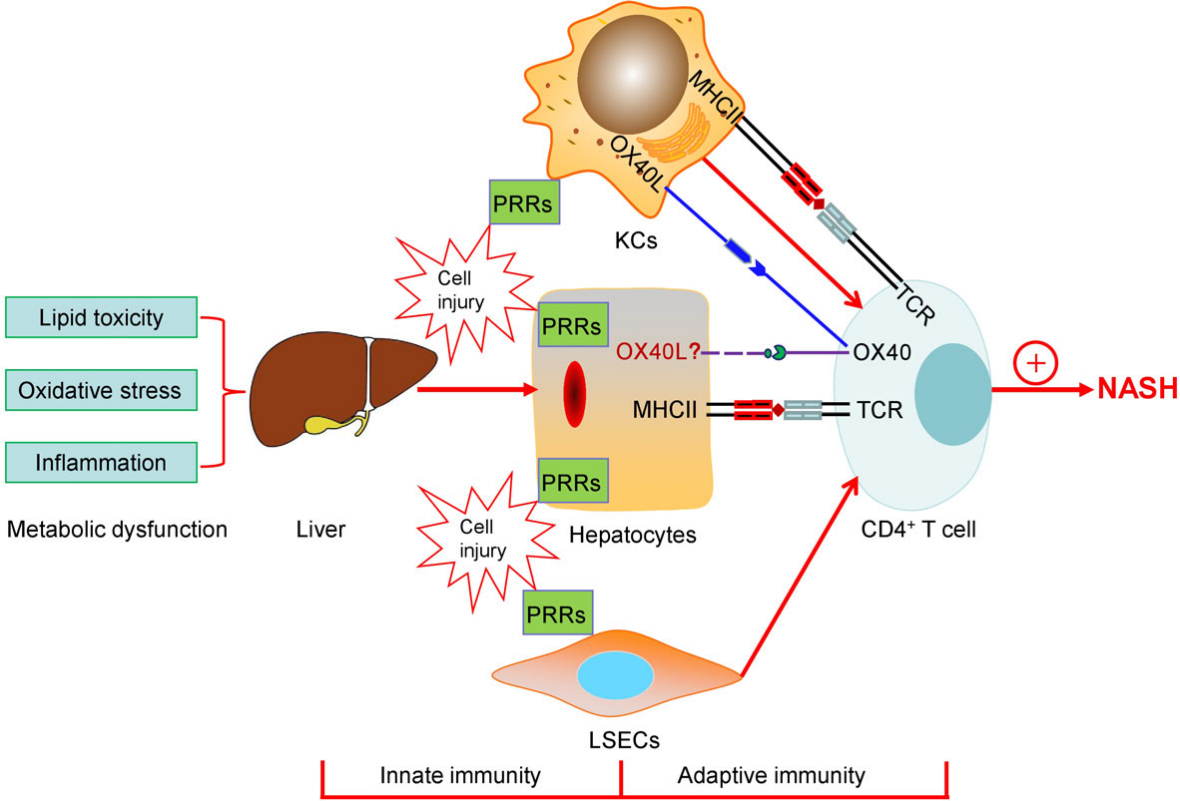
The schematic diagram of both innate immunity and adaptive immunity contributing to the progression of NASH. Lipid toxicity, oxidative stress, and inflammation may give rise to the injuries of hepatocytes, macrophages (KCs), and liver sinusoidal endothelial cells (LSECs), where PRRs including TLRs and NLPR3 inflammasome sense the signals through both pathogen- and danger-associated molecular patterns and trigger proinflammatory responses. KCs act as a bridge between the innate and the adaptive immune responses. The roles of LSECs in NASH are not discussed in the present paper. Hepatocytes as APCs may play a role in T cell-mediated adaptive immunity and express the MHCII molecules, which are elevated during NASH, providing the first signal for CD4^+^ T cell activation. Simultaneously, T cell costimulatory signal pathways, such as OX40-OX40L, which may mediate the cross-talk between hepatocytes and T-cells, are associated with the progression of NASH. Finally, CD4^+^ T cells are involved in the pathogenesis and development of NASH.

Chronic inflammation plays a critical role in NASH. Increasing evidence has indicated that both innate immunity and adaptive immunity contribute to the progression of NASH (**Figure 1**). Lipid toxicity, oxidative stress, and inflammation may give rise to the injuries of hepatocytes, macrophages (KCs), and liver sinusoidal endothelial cells (LSECs), where PRRs including TLRs and NLPR3 inflammasome sense the signals through both pathogen- and danger-associated molecular patterns and trigger proinflammatory responses. The KCs act as a bridge between the innate and adaptive immune responses. The role of LSECs in NASH is not discussed in this review due to the length limit of the article. Although it is still unclear whether hepatocytes provide T-cell costimulatory signal, they express the MHCII molecules and may act as nonclassic APCs contributing T cell-mediated adaptive immunity and liver fibrosis in NASH. In the past decade, a large body evidence demonstrates that CD4^+^ T cells are critically involved in the pathogenesis and progression of NASH. Different CD4^+^ T cell subsets exhibit diverse effects on liver fibrosis. Th1 and Th17 cells are proinflammatory and profibrogenic, while the role of Th2 cells in hepatic fibrosis is complicated. Th22 and Treg cells may be both anti- and/or pro-fibrotic depending on disease setting and the stage of the disease.

Although great progress has been made in demonstrating the mechanism of the chronic inflammation and functions of immune cells particularly the CD4^+^ T cell subsets in NASH, many critical questions remain unanswered. With the help of modern techniques including single-cell or single-nucleus RNA sequencing combined with interactive analysis, we should be able to gain more insights into the underlying cellular and molecular mechanisms of NASH and identify new potential therapeutic targets for treating liver fibrosis. Finally, it is noteworthy to emphasize the differences in compositions and subsets of immune cells in the livers between human and mice. Thus, mice or rats with humanized immune system are urgently needed for future study on the role of immune cells and inflammation in NASH.

## Author contributions

YZ: wrote the manuscript. YZ, HZ and YY: analysized the data. YZ, XZ, FZ and YG: revised the manuscript. All authors contributed to the article and approved the submitted version.

## Funding

This work was supported by the National Natural Science Foundation of China (No. 81970606, 81970595, 81970636 & 81970642); the Shenzhen Basic Research Project (No. JCYJ20210324095005015).

## Conflict of interest

The authors declare that the research was conducted in the absence of any commercial or financial relationships that could be construed as a potential conflict of interest.

## Publisher’s note

All claims expressed in this article are solely those of the authors and do not necessarily represent those of their affiliated organizations, or those of the publisher, the editors and the reviewers. Any product that may be evaluated in this article, or claim that may be made by its manufacturer, is not guaranteed or endorsed by the publisher.

## Supplementary material

Supplementary Figure 1 The changes of immune cell subsets in the livers between humans **(A)** and mice **(B)** by single cell RNA-sequencing (scRNA-seq) analysis. We analyzed and specifically focused on the immune cells from publicly available liver single-cell transcriptome datasets of humans and mice. The proportions of CD4^+^ T cells, CD8^+^ T cells, NK T cells, NK cells, γδ T cells, TEMs, and monocytes in murine livers were relatively less than those in humans, but the numbers of B cells, DC cells, and KCs were more in mice than those in humans. The original data from the database in the *www. livercellatlas.org* were downloaded with the permission of the authors. Notes: TEMs, effector memory T cells; KCs, Kupffer cells.
