# Supplementary figures and images for: CD4+ T cell activation and inflammation in NASH-related fibrosis
Source: Front Immunol. 2022 Aug 10;13:967410. doi: 10.3389/fimmu.2022.967410 (PMC9399803; doi:10.3389/fimmu.2022.967410)

**A**

## Humans

### Lymphoid cells

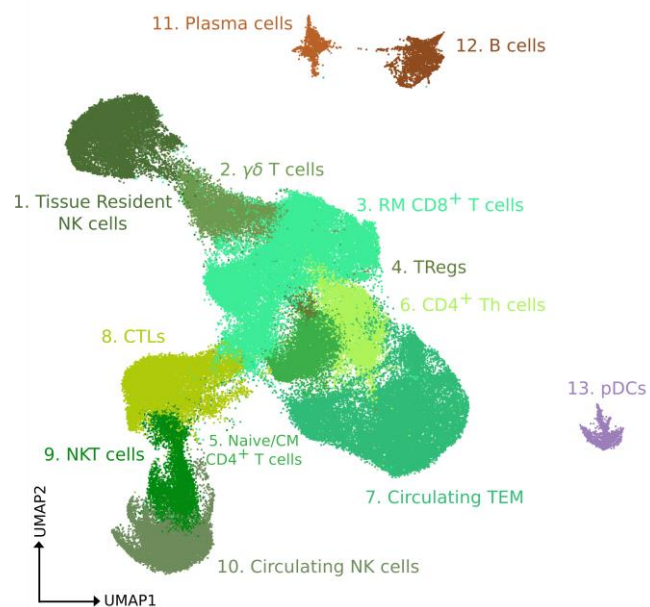

### Myeloid cells

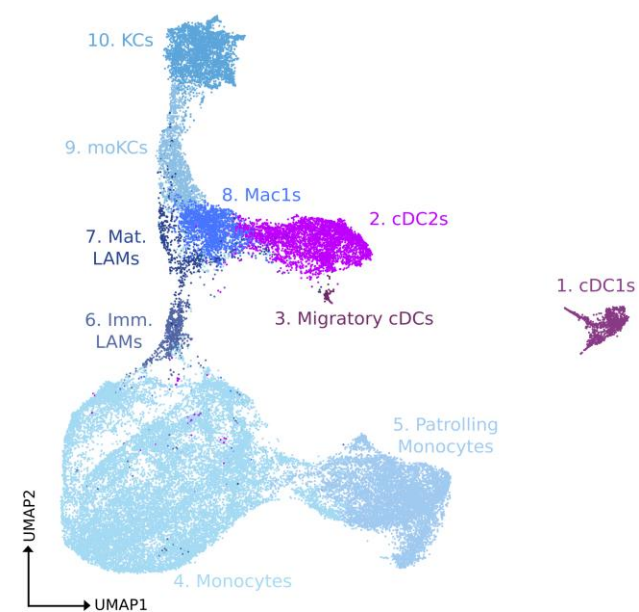**B**

## Mice

### Lymphoid cells

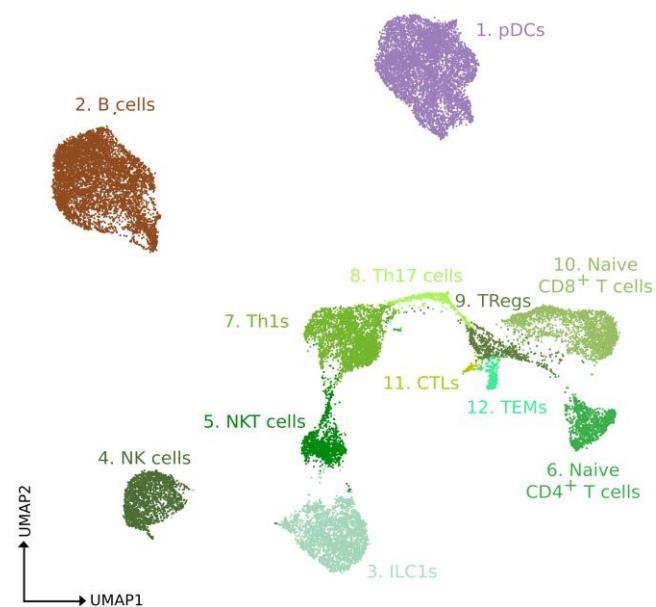

### Myeloid cells

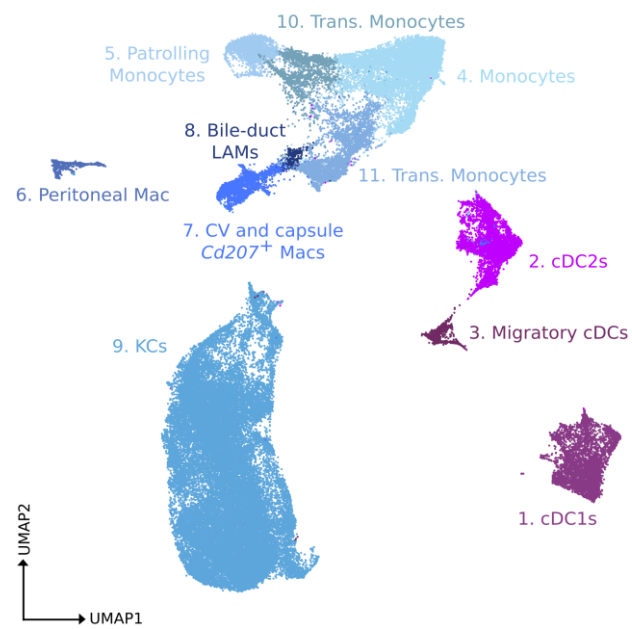

Supplement: Supplementary Figure 1 — The changes of immune cell subsets in the livers between humans (A) and mice (B) by single cell RNA-sequencing (scRNA-seq) analysis. We analyzed and specifically focused on the immune cells from publicly available liver single-cell transcriptome datasets of humans and mice. The proportions of CD4+ T cells, CD8+ T cells, NK T cells, NK cells, γδ T cells, TEMs, and monocytes in murine livers were relatively less than those in humans, but the numbers of B cells, DC cells, and KCs were more in mice than those in humans. The original data from the database in the www. livercellatlas.org were downloaded with the permission of the authors. Notes: TEMs, effector memory T cells; KCs, Kupffer cells. [file DataSheet_1.pdf]
